# Improving tribological and anti-bacterial properties of titanium external fixation pins through surface ceramic conversion

**DOI:** 10.1007/s10856-016-5816-0

**Published:** 2016-11-24

**Authors:** Huan Dong, Tatiana Mukinay, Maojun Li, Richard Hood, Sein Leung Soo, Simon Cockshott, Rachel Sammons, Xiaoying Li

**Affiliations:** 1 Royal Derby Hospital, Derby, DE22 3NE UK; 2 School of Metallurgy and Materials, University of Birmingham, Birmingham, B15 2TT UK; 3 School of Engineering, University of Birmingham, Birmingham, B15 2TT UK; 4 School of Dentistry, University of Birmingham, Birmingham, B4 6NN UK

## Abstract

In this study, an advanced ceramic conversion surface engineering technology has been applied for the first time to self-drilling Ti6Al4V external fixation pins to improve their performance in terms of biomechanical, bio-tribological and antibacterial properties. Systematic characterisation of the ceramic conversion treated Ti pins was carried out using Scanning electron microscope, X-ray diffraction, Glow-discharge optical emission spectroscopy, nano- and micro-indentation and scratching; the biomechanical and bio-tribological properties of the surface engineered Ti pins were evaluated by insertion into high density bone simulation material; and the antibacterial behaviour was assessed with *Staphylococcus aureus* NCTC 6571. The experimental results have demonstrated that the surfaces of Ti6Al4V external fixation pins were successfully converted into a TiO_2_ rutile layer (~2 μm in thickness) supported by an oxygen hardened case (~15 μm in thickness) with very good bonding due to the in-situ conversion nature. The maximum insertion force and temperature were reduced from 192N and 31.2 °C when using the untreated pins to 182N and 26.1 °C when the ceramic conversion treated pins were tested. This is mainly due to the significantly increased hardness (more than three times) and the effectively enhanced wear resistance of the cutting edge of the self-drilling Ti pins following the ceramic conversion treatment. The antibacterial tests also revealed that there was a significantly reduced number of bacteria isolated from the ceramic conversion treated pins compared to the untreated pins of around 50 % after 20 h incubation, *P* < 0.01 (0.0024). The results reported are encouraging and could pave the way towards high-performance anti-bacterial titanium external fixation pins with reduced pin-track infection and pin loosing.

## Introduction

External fracture fixation is a common orthopaedic procedure that is used increasingly in a variety of trauma settings. These include highly comminuted fractures, open fractures, fractures associated with gross soft tissue damage or in the multiply injured patient. This method of fixation can be used either as a temporising measure before definitive internal fixation or as definitive treatment itself. External fixation entails the use of percutaneously placed transosseous pins and/or wires secured to external scaffolding to provide support to a limb. For example, an Ilizarov frame can provide stability to the fracture, protect skin grafts, and allow access to adjacent soft tissue. These percutaneous K-wires cause minimal disruption of the blood supply and soft tissues through the zone of injury [1]. Now, external fixation has evolved from being used primarily as a last resort fixation method to becoming a main stream technique used to treat a myriad of bone and soft tissue pathologies.

A well designed pin is essential to ensure a solid fixation, ease of insertion and reduce risk of complications. Stainless steel and titanium self-drilling/self-tapping Schanz pins offer a one-step insertion where pre-drilling is not required because the self-drilling tip acts like a new, sharp drill bit. Combined with the unique cutting geometry, this one-step procedure allows reduced insertion time and temperature for optimal performance [2].

Austenitic stainless steels have been the material of choice for external fixation pins owing to their attractive combination of excellent corrosion resistance, good mechanical properties and adequate biocompatibility coupled with their outstanding formability and cost-effectiveness [3]. However, pin track infection is a common complication in stainless steel external fixation systems with infection rates ranging from 2 % to as high as 30 % [4]. This is because the pin entry site is constantly open to the environment and presents a critical interface between the external stainless steel pin and internal soft tissues and bone. The critical consequences of infected pin sites are pin loosening, fracture destabilisation and osteomyelitis, thus leading to additional surgical interventions and delayed or non-union [5]. Indeed, how to combat multi-resistant bacteria has become one of the greatest challenges in the treatment of post-operative infections.

Antibacterial or self-disinfecting surfaces have progressively become a primary strategy in the fight against medical device-associated infections [6]. Antibacterial surfaces can be created by coating surfaces with silver since silver and its compounds have a broad-spectrum of antimicrobial activities. For instance, pure Ag has been used to coat stainless steel surfaces. However, it has been reported that no statistically significant difference in the clinical outcomes of Ag-coated and uncoated steel fixation pins could be observed [7]. Some attempts have also been made to develop Ag-containing nanocomposite coatings with increased hardness [5]. However, there is a lack of controlled release kinetics from the Ag-containing coatings. The major concerns over Ag-containing composite coatings under tribological application are how to ensure adequate bonding between the coating and the substrate. Failure of such Ag-containing coatings will lead to fast leaching of Ag ions and may result in toxicity [8].

Alternatively, the track infection caused by stainless steel pins could be partially addressed by replacing stainless steel with titanium and titanium fixation pins are now commercially available. This is mainly because the TiO_2_ oxide layer formed on titanium and its alloys has shown some extent of anti-bacterial efficacy [9]. Some researchers have attempted to further increase the anti-bacterial efficacy of Ti and its alloys by coating their surfaces with an antibacterial coating [10, 11].

Anodising has long been used to form a thin (<0.1 µm) and compact TiO_2_ film on Ti surfaces when the anodic oxide is insoluble in the electrolyte. More recently, TiO_2_ nanotube arrays are generated when the anodic oxide is moderately soluble in the electrolyte, which showed a high antibacterial efficacy [12]. However, their durability under wear conditions is low in view of their limited thickness and low hardness (373HV) [13]. As a wet chemical surface coating technology, sol–gel is employed by some researchers to produce a dense and smooth anti-bacterial TiO_2_ layers (<10 µm) on Ti surfaces without affecting the surface morphology at the micrometric scale. However, this technique is not an economic solution to complex 3D surfaces due to dip or spray coating, the bonding to the substrate is normally low, and cracking is a major technical challenge for sol–gel coating [14].

A thick TiO_2_ coating can be produced by plasma electrolytic oxidation (<40 µm) or thermal spraying (100–400 µm). These two techniques present several advantages such as high deposition rate, relatively low-cost and possibility for doping with metal nanoparticles (such as Ag). However, it is difficult, if not impossible to produce dense and pure TiO_2_ coating with a smooth surface by these two techniques [15].

As for all coating technologies, the bonding of these coatings to titanium substrate is a major concern for such bio-tribological devices as self-drilling/self-tapping Schanz pins. In addition, it is well-known that titanium and its alloys are characterised by poor mechanical properties in terms of low hardness and low load bearing capacity. Therefore, without strong mechanical support from the substrate, the very thin (nanometric) TiO_2_ films formed in air at room temperature [16] or the anti-bacterial coatings produced by the above coating methods can be damaged by tribological interaction due to the so-called “thin egg-shell effect” [17]. This led to the loss of the anti-bacterial property and costly duplex treatments were developed to increase the durability of thin antibacterial coatings [18]. In addition, Ti alloys have reputation of poor tribological properties in terms of low hardness, high friction and strong adhesive wear [19, 20]. Therefore, from a bio-mechanics point of view, the wear of Ti self-drilling tip or cutting edge necessitates an increased insertion force [1]. This will in turn result in increased temperature at bone-pin surface during insertion, which would cause damage to the bone and retard its healing after operation [21].

To this end, the present paper reports an novel approach to generate a multi-functional surface for titanium Schanz pins by converting their Ti surfaces into a TiO_2_ ceramic layer through thermal oxidation of Ti in an oxygen-containing atmosphere. In the meantime, oxygen diffuses into the subsurface to form a hard oxygen diffusion hardened case. This oxygen diffusion hardened case can provide a strong mechanical support to the surface TiO_2_ ceramic layer, thus effectively conferring a high load bearing capacity of the CCT treated Ti surafces. Most importantly, the bonding between the surface ceramic layer and the oxygen diffusion hardened subsurface is strong due to the in-situ conversion nature of the formation of the TiO_2_ ceramic layer. In addition, this ceramic conversion treatment (CCT) is fully environmentally friendly and cost-effective. This is because unlike sole-gel, anodising or plasma electrolytic oxidation, neither chemical precursors nor toxic by-products are involved in the CCT; unlike thermal spraying, all the 3D surfaces with a complex geometry can be treated.

This CCT was developed from University of Birmingham [22], [23] and has been successfully used for motor sports for tribological property enhancement [24]. However, the potential of this CCT technique has not been explored for medical devices. This can pave the way towards high-performance anti-bacterial titanium external fixation pins because the CCT process could significantly increase their surface hardness and load bearing capacity, significantly reduce their friction and wear, and confer high anti-bacterial efficacy to their surfaces.

## Materials and methods

### Substrate materials

Ti6Al4V coupon samples with a thickness of about 5 mm were cut from hot rolled and annealed bar (supplied by IMI Titanium Ltd) of 25.4 mm (i.e., 1 inch) in diameter for ceramic conversion treatments. The treated coupon samples were used for microstructure and properties characterisation. Commercial Ti6Al4V titanium alloy self-drilling/self-tapping pins (Apex 5018-6-150S) were used for ceramic conversion treatment under optimised conditions derived from the characterisation of CCT treated coupon samples. The diameter of the thread/shaft, total length and thread length were 5, 150 and 50 mm respectively.

### CCT

Coupon samples were carried out the treatments between temperatures of 500 to 850 °C for times of 10 to 100 h. The treatments were carried out in a controlled atmosphere containing 20 % oxygen and 80 % nitrogen under 100 Pa to convert the titanium surface into an oxide layer and to introduce oxygen into the subsurface to form an oxygen solid solution hardened diffusion case. The treatment temperature and time are two important factors affecting the surface layer quality and tribological performances, thus the treatment atmosphere and the pressure were kept constant. Based on the systematic optimisation work on the coupon samples a patented [22, 23] treatment conditions of 600 °C, 85 h was selected for the titanium alloy pins.

### Characterisation of CCT treated coupons and the pins

The microstructure of CCT treated Ti6Al4V coupon samples was fully characterised using glow discharge optical emission spectrometry (GDOES, GDA650HR, Spectrma) to measure the composition depth profiles of oxygen, titanium, vanadium, and aluminium. A Philips PW 1050 X-ray spectrometer (with Cu Κ_α_ radiation) was used to identify the phases present in the treated specimens. A Jeol 6300 SEM equipped with an EDX facility was used to examine the cross-sectional microstructure of the CCT treated Ti6Al4V surface. Surface layer microstructure and phase composition of the 600 °C, 85 h treated sample was examined by transmission electron microscope (TEM) using a JEOL 4000FX. The TEM specimens were prepared perpendicular to the surface (XTEM). Two slabs of surface treated samples were cut and glued together as a ‘sandwich’. This sandwiched sample was cut cross-sectional into 1 mm thickness slices and they were then mechanical thinned, dimpled and finally ion-beam milled to penetration.

The mechanical properties in terms of hardness, Young’s modulus of the surface oxide layer formed by CCT were probed using a Vantage Nano indentation machine with the typical resolutions of 0.1 nm in displacement and 100 nN in force. The hardness profile of treated Ti6Al4V was obtained on polished metallographic sections normal to the surface by a Leitz Miniload hardness tester with a Knoop indenter under a load of 25 gf (0.24 N). In view of the scatter of the microhardness data, especially in two phase microstructure regions, the points plotted was a mean of five measurements made at the same depth with the standard deviation as the error bars.

The adhesion of the surface oxide layer to the substrate was studied by friction monitored scratch testing using a Teer ST-200 scratch tester. During the test a load was applied to the Rockwell’C’ spherical cone diamond indenter which increased linearly at a loading rate of 20 N /min, the table moved at the speed of 4 mm/min. The load at which the friction force rapidly increases, corresponding to the delamination of the surface oxide layer, is defined as the critical load. The failure mechanism was further studied by SEM observation.

### Insertion test in bone simulation material

**Fig. 1:**
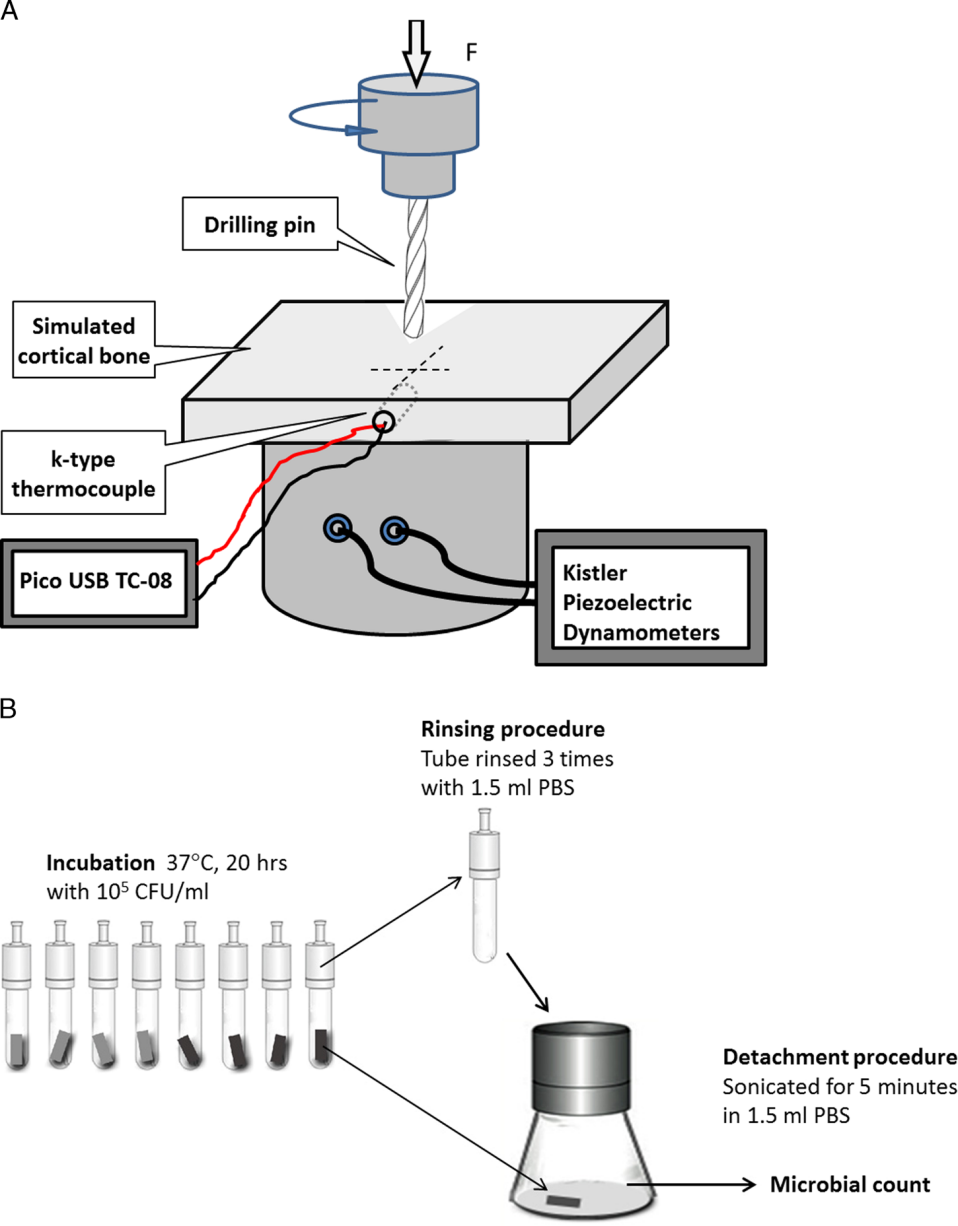
A schematic of **a** the pin insertion set up and **b** experimental method for the assessment of the antimicrobial efficacy of insertion Ti pins

The biomechanical and bio-tribological properties of CCT treated Ti6Al4V pins were evaluated by insertion tests using bone simulating material. This is mainly because the mechanical properties (such as hardness and wear) of fresh animal bones depend highly on the type, the position and the freshness of the bones. Hence, it is difficult, if not impossible, to compare the insertion behaviour of untreated and treated titanium pins using a fresh animal bone. Therefore, SAWBONES^®^ (Europe AB, Sweden) high-density short fibre filled resin (density 2.5 %, compressive strength, 157 MPa) bone substitute blocks (30 x17 x 4 mm) were used to simulate pin insertion into cortical bone. An industrial drilling machine (Mazak Vertical Centre Smart 430A) was employed to conduct the insertion test under a constant feed rate (i.e., insertion speed) of 25 mm/min. As schematically shown in Fig. 1a, a bone simulating block was held in a fixture, which connected to a Kistler Piezoelectric Dynamometers (Type 9273) for the measurement of axial insertion force. A k-type thermocouple was placed in a 1 mm in dimeter and 2 mm deep hole of the insertion material, situated at a distance of 5 mm from the axis of the inserted pin, connected with the Pico USB TC-08 data logger.

The pin was inserted a distance of 23 mm without pre-drilling through the plate at a rotational speed of 50 rpm. The applied axial force needed to achieve this fixed insertion speed and the temperature increase during insertion were continuously recorded by the Kistler Piezoelectric Dynamometers and the Pico USB TC-08 data logger, individually. The insertion performance was evaluated by determining the maximum axial insertion force and the maximum temperature increase during insertion. Post-insertion examination was conducted to identify the wear of CCT treated and untreated pins using both Toolmakers optical microscopy and FEI Quanta 3D FEG FIB-SEM.

### Antibacterial test

Based on the method of Furkert et al. [7], the antibacterial activity of untreated and CCT treated Ti6Al4V pins 5 mm in diameter and 8 mm in length cut from the head of the fixation pins (*n* = 4), were tested with *Staphylococcus aureus* (*S. aureus*) NCTC 6571. The bacteria were cultured in sterile tryptone soya broth (Oxoid, UK) overnight at 37 °C in a shaking incubator at 100 rpm. Serial dilutions were made to achieve a suspension of 10^5^ colony forming units per ml (cfu/ml) in a sodium chloride peptone broth pH 7.0 (SCPB) mix. Four untreated (control) and four CCT treated Ti6Al4V pins (5 mm diameter, 8 mm length), were sterilised by autoclaving (120 °C, 1 bar pressure) and then completely immersed in 1.5 ml of the bacterial suspension in individual sterile microfuge tubes. The tubes were incubated at 37 °C for 20 h without shaking.

Following incubation the bacterial suspension was discarded from the tubes containing the fixation pins and they were rinsed three times with 1.5 ml of sterile Phosphate-buffered saline (PBS), to remove low surface-adherent bacteria. The samples were then sonicated for 5 min in 1.5 ml PBS in a VitaSonic In-ceram sonic bath to detach any high surface-adherent bacteria. 1 ml of this suspension was then diluted 10^−2^ in PBS and 0.1 ml aliquots were plated in triplicate on TSA to determine the viable counts of the recovered bacteria. The results are expressed as the mean ± standard deviation (SD). A schematics of the experimental method for the assessment of the antimicrobial efficacy of insertion Ti pins is shown in Fig. 1b.

## Results

### Microstructure characterisation of CCT treated coupon samples

**Fig. 2:**
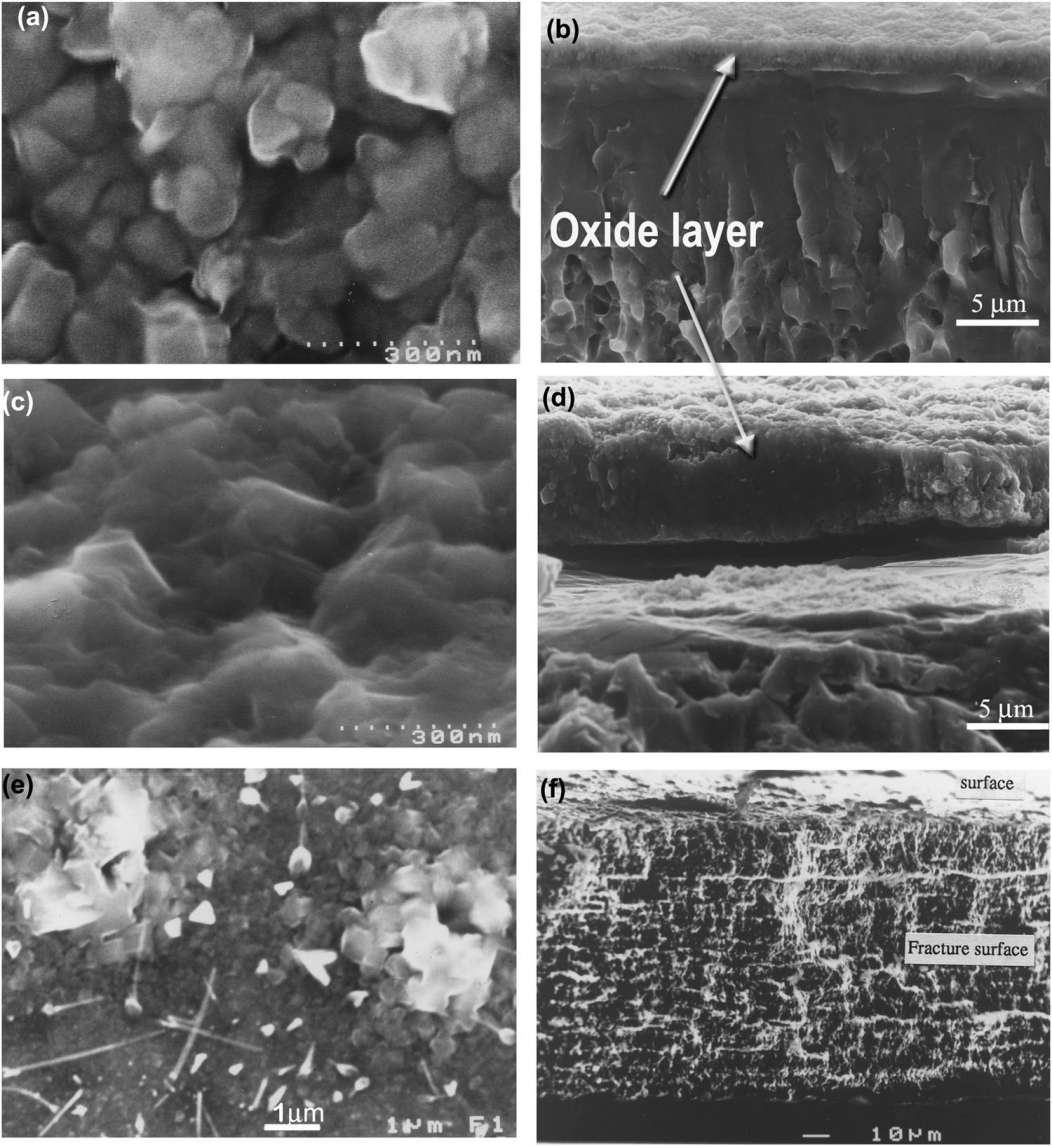
Typical SEM images of surface morphology (left) and fractured cross-sectional (right) microstructures of the CCT treated Ti6Al4V samples: (**a**, **b**) 600 °C, 85 h; (**c**, **d**) 700 °C, 20 h and (**e**, **f**) 800 °C, 20 h

**Fig. 3:**
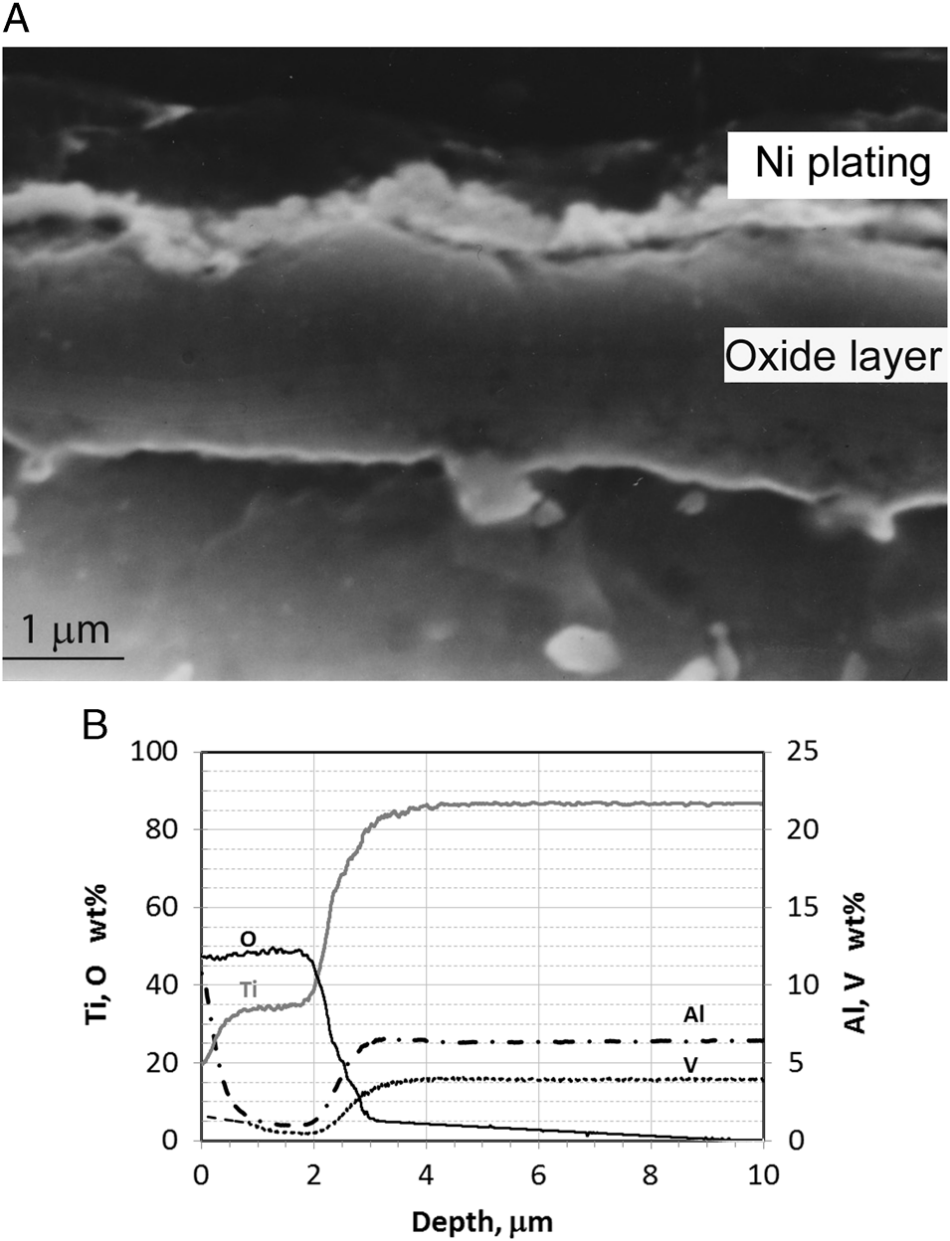
**a** SEM image of cross-sectional layer structure and **b** GDOES depth chemical composition profiles of optimal CCT treated (600 °C, 85 h) Ti6Al4V alloy

**Fig. 4:**
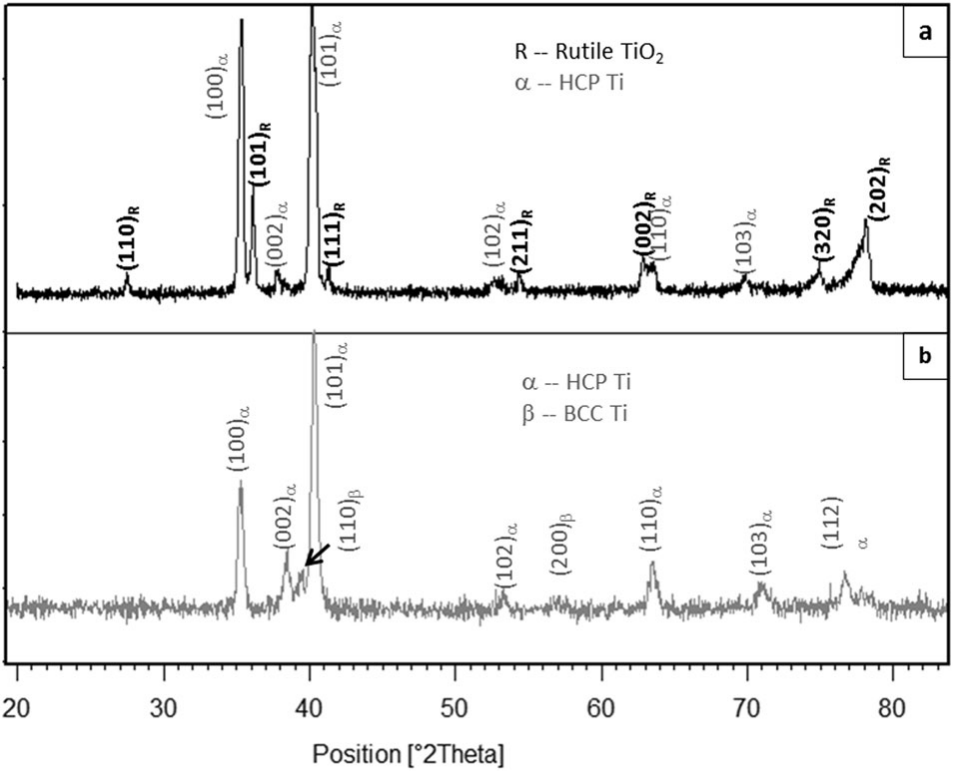
XRD patterns of **a** CCT treated and **b** untreated Ti6Al4V alloy, showing formed rutile TiO_2_ on the treated sample

**Fig. 5:**
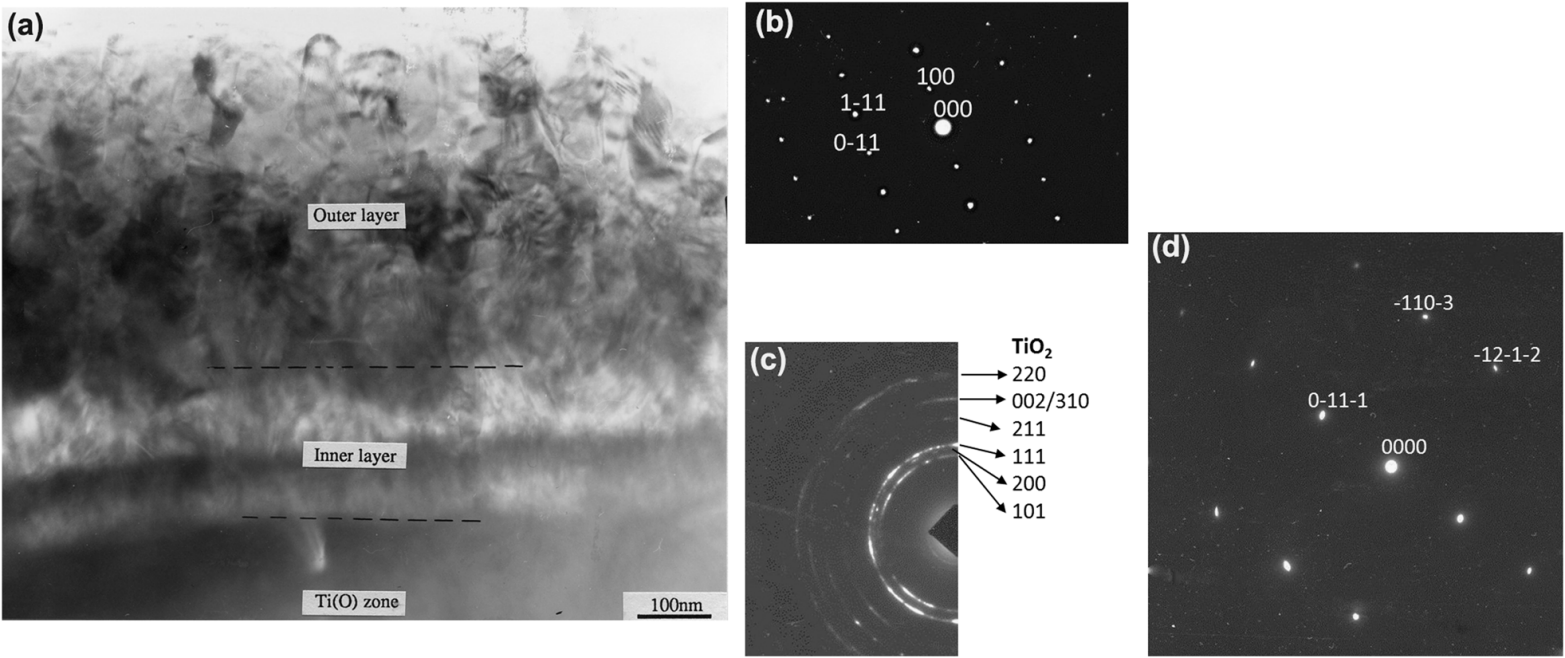
XTEM microstructure **a** of the surface oxide layer and corresponding SAD patterns taken from **b** an outer layer TiO_2_ grain, **c** inner layer of fine columnar TiO_2_ grains and **d** substrate diffusion zone of *α*-Ti(O)

Typical SEM images of surface morphology and fractured cross-sectional microstructures of the CCT treated Ti6Al4V samples are shown in Fig. 2. It was observed that the specimens were all covered by oxides. When treated at 550 °C (up to 480 h) and 600 °C (up to 85 h), the oxides are smooth and fine roundish shaped, as is typified in Fig. 2a. When temperatures increased to 700 °C, less than 10 h treatment resulted a rough surface of the specimen with sharp-angled oxide particles (see Fig. 2c). Further increase the treatment temperatures over 800 °C, less than half an hour treatment, the surface of the specimen was coved by blistered large granular and whisker oxides as evidence in Fig. 2e. SEM images of Fig 2b, d and f revealed the oxide layer structure and the bonding state of 600, 700 and 850 °C treated samples. It can be clearly seen that oxides formed at 600 °C are dense, well bonded/adhered to the substrate. Conversely, stratification of the oxide layer and weak bond to the substrate were observed when samples treated at 700 °C, as revealed in Fig. 2d. Pronounced stratification and spallation of the oxide layer from 800 °C and above treated samples was observed, which was caused by the interfacial stresses [25]. Based on the above observation, treatment conditions in terms of temperature and time was chosen as 650 °C and 85 h. Further microstructure characterisation detailed in the rest part of this section is for the coupon samples treated under this condition. Figure 3a reveals the cross-sectional layer structure of the optimal CCT treated sample and the corresponding GDOES composition-depth profiles is shown in Fig. 3b. High amount of oxygen was introduced to the surface and levelled within 2 um depth from the surface before a considerable drop in the diffusion zone (Fig.3b). The X-ray diffraction (XRD) patterns of the CCT treated and untreated Ti6Al4V samples are shown in Fig. 4 and it can be seen that the XRD pattern of the untreated material (Fig. 4b) is dominated by the alpha Ti peaks with a few weak peaks for beta Ti. After CCT treatment, some additional peaks appeared which can be identified as TiO_2_ rutile (Fig. 4a). The relative intensity of these rutile peaks is much lower than that of alpha because the X-ray penetration depth is estimated to be about 10 μm, which is much larger than the thickness of the surface rutile layer (~2μm). In addition, no peaks from beta Ti were identified mainly because of the inward diffusion of the alpha stabiliser oxygen. XTEM observation revealed features of the surface oxide layer structure as shown in Fig. 5. It can be seen that the oxide layer composed of two sublayers: an inner layer of fine columnar grains, as evidence by ring patterns (Fig. 5c) and an outer layer of well-growing equal-axis grains. Analysis of the Selected area diffraction (SAD) patterns taken from inner and outer sublayers identified the rutile TiO_2_ phase, as indexed in Figs. 5b,c. The SAD pattern taken from the adjacent substrate (Fig. 5d), revealed an expanded HCP structure of α-Ti(O). The interface between the oxide layer and the adjacent oxygen diffusion zone, appeared dense and without voids.

### Mechanical and tribological properties of CCT treated coupon samples

**Fig. 6:**
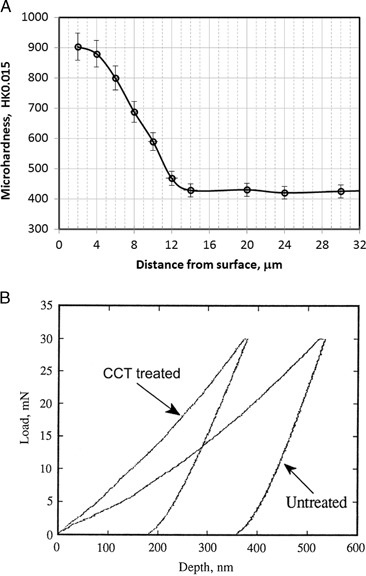
**a** Knoop harness profile cross the diffusion zone for 600 °C, 85 h CCT treated sample **b** nano-indentation load-unload verses depth hysteresis curves for as-received and CCT treated specimens

Microhardness profile cross the diffusion zone for 600 °C, 85 h treated sample is shown in Fig. 6a and the thickness of the hardened layer was estimated from the profile to be 15 µm with a near-surface hardness of about 900HK_0.015_, which is about three times that of untreated material. This is because in addition to the formation of a thin surface oxide layer about 2 µm (Fig. 3a), an oxygen diffusion zone was also formed during the CCT treatment and produced effective solid solution hardening by the oxygen [23].

Because the surface oxide layer is very thin, nano-indentation was used to probe the mechanical properties of the surface oxide layer. The load-unload vs. depth hysteresis curves recorded during the nanoindentation tests for as-received and CCT treated specimens are shown in Fig. 6b. It can be seen clearly that the CCT treated sample exhibits a much shallower penetration depth and a higher elastic recovery than the as-received sample. The nano-hardness (H) and the Young’s modulus (E) of the rutile oxide layer measured to be 10.5 ± 0.5 GPa and 146.7 ± 1.2 GPa, respectively, while for the as-received sample, the H and E are 4.3 ± 0.4 GPa and 114.3 ± 1.3 GPa, respectively. The ratio of H/E is increased from 0.029 for untreated sample to 0.071 for the CCT treated sample. This indicates that compared with the untreated material the rutile oxide layer possessed a much higher capacity for elastic deformation and hence a reduced tendency for adhesive wear and scuffing [23].

**Fig. 7:**
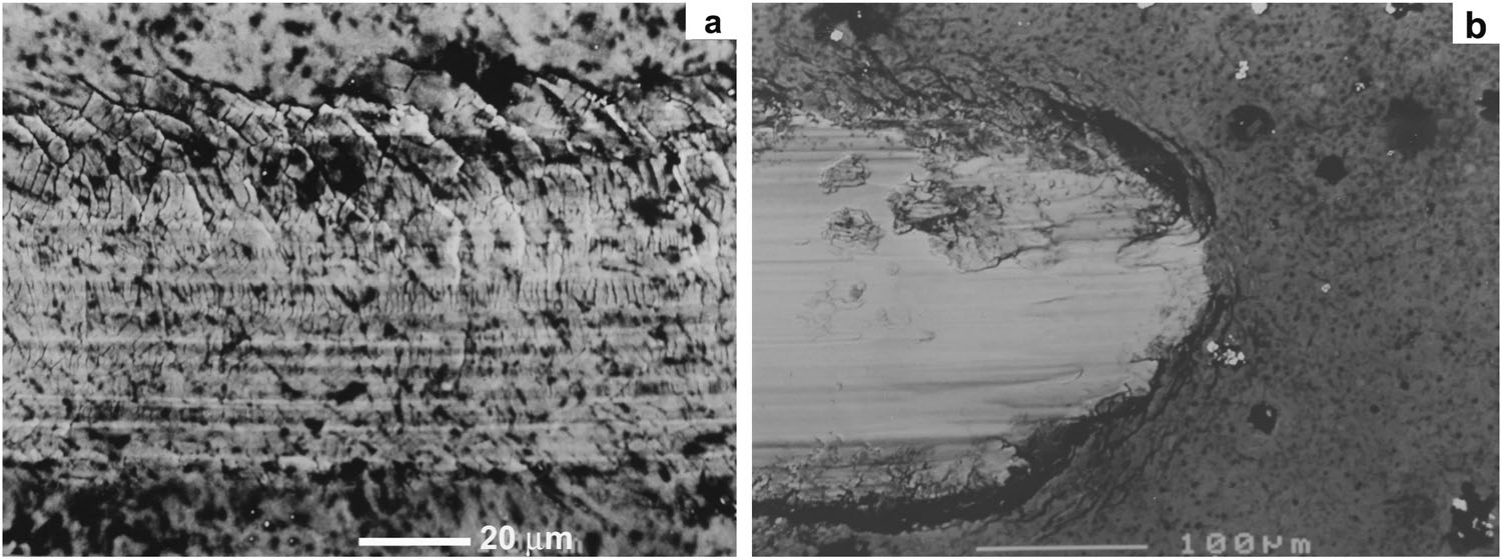
SEM BS images showing **a** typical conform cracking and **b** neither flaking nor spallation at the end of the scrach

Scratch tests carried out on the CCT treated samples revealed that no clear cracking occurred at the beginning; then, conformal cracking started to occur when the load increased to about 40 N (Fig. 7a); but the oxide layer remained adherent to the substrate without flaking until it was entirely removed when the critical load about 70.5 ± 2.2 N was exceeded and the monitored friction increased rapidly. Even at the end of scratch test where severe pile-up of the substrate material is clearly evidenced, neither flaking nor spalling was appreciable (Fig. 7b). This indicates that the oxide layer formed by in-situ conversion remains adherent and failure was mainly caused by excessive plastic deformation in the substrate [26].

### Biomechanical properties of the self-drilling/self-tapping pins

**Fig. 8:**
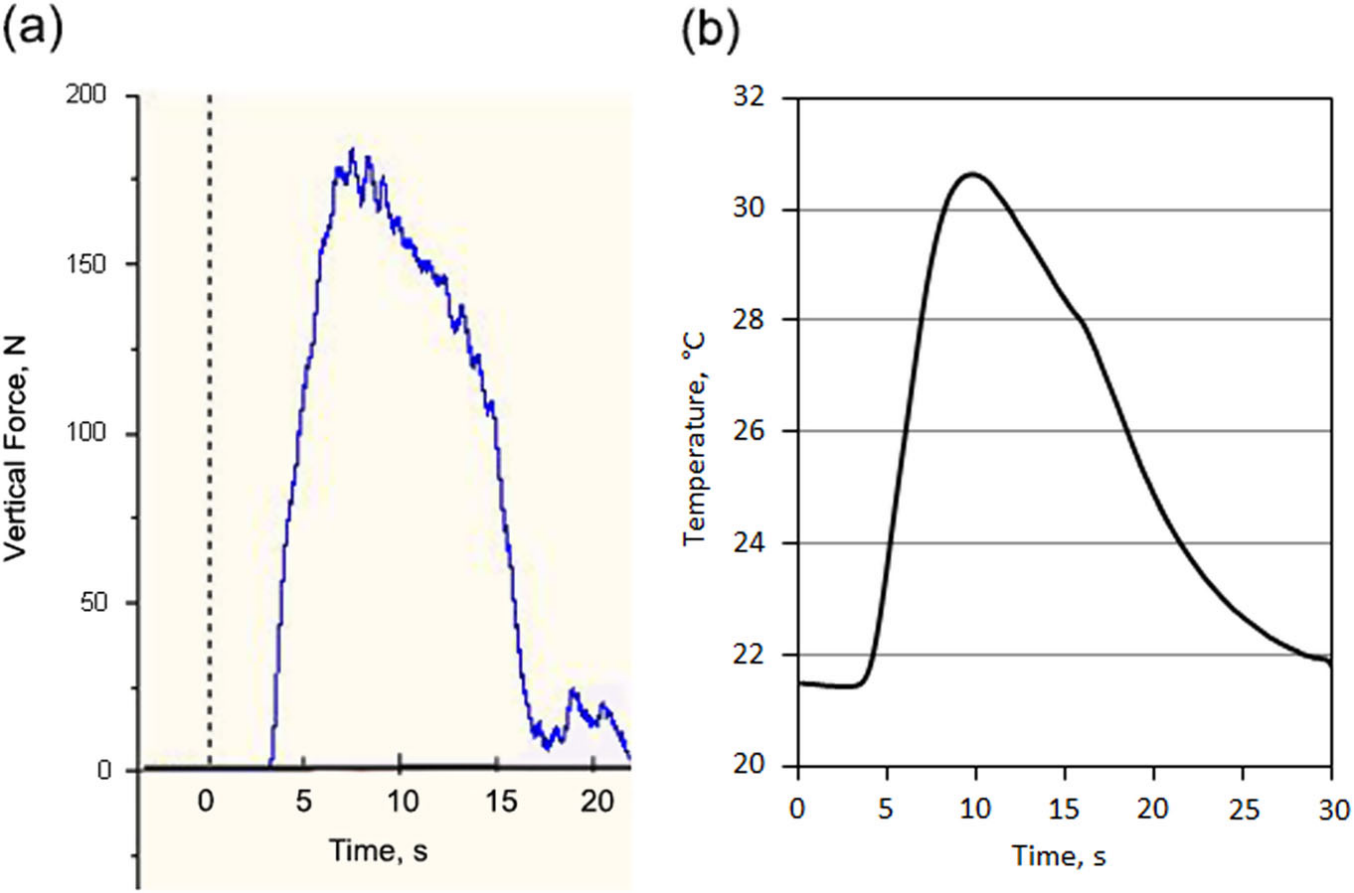
Recorded **a** maximum axial force and **b** temperature change against the insertion time under the fixed insertion speed of 25 mm/min to SAWBONES^®^ cortical bone plates

**Fig. 9:**
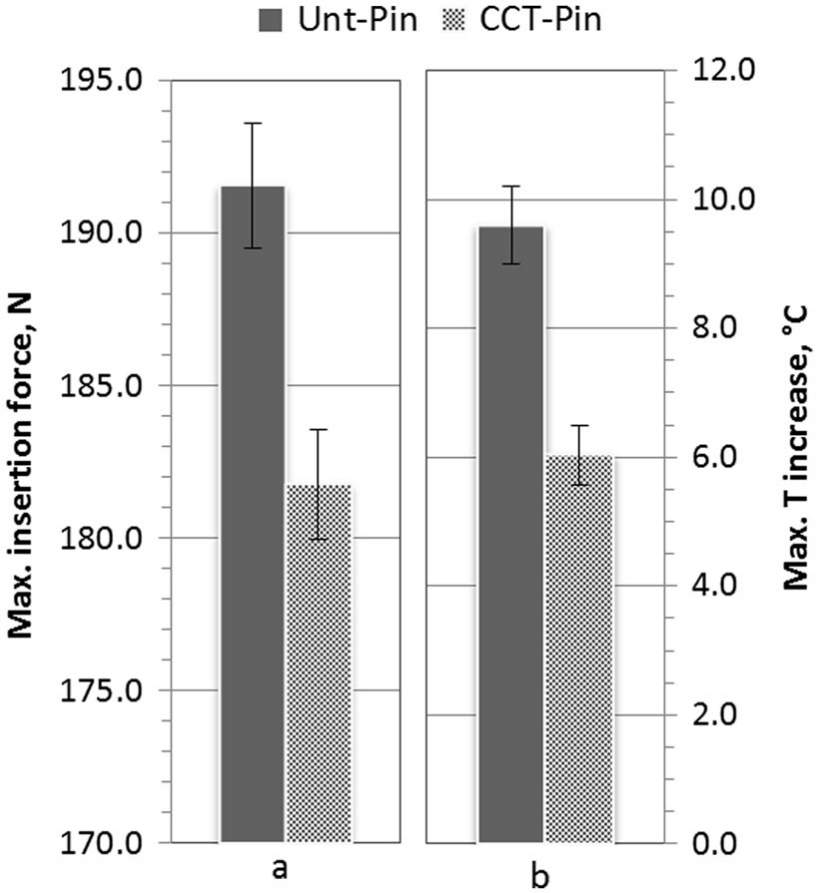
Comparison of **a** maximum insertion forces and **b** maximum temperature increase for untreated and CCT treated self-drilling/self-tapping pins inserting to SAWBONES^®^ cortical bone plates

The typical recorded axial force needed to achieve the insertion distance of 23 mm under the fixed insertion speed of 25 mm/min as a function of the insertion time is exemplified in Fig. 8a. It can be seen that the applied axial force increased almost linearly and smoothly before it reached the maximum value when the self-drilling/self-tapping pin was driven into the bone simulation material; then it reduced relatively slowly with some fluctuation when the pin went through the bone simulation block; and finally it rapidly reduced before it flattened at a low value after the pin fully went through the block. The temperature variation during the insertion tests was recorded by the Kistler Piezoelectric Dynamometers and the Pico USB TC-08 data logger, which is shown in Fig. 8b. It is revealed that the temperature of the simulated cortical bone started to increase after about 5 s and reached to a maximum value at the insertion time of 10 s and then reduced slowly to room temperature after about 30 s. Compare with the axial force variation during the insertion, it can be seen that although the trends are the same, the recorded temperature showed hysteresis in relative to the insertion force recorded. This phenomenal is largely caused by the thermal transfer in the bone simulation material as the thermal couple was set at a distance of 5 mm from the axis of the inserted pin (Fig. 1a) with a small gap of 2.5 mm between the tip of the thermal couple and the surface of the pin. The most important biomechanical characteristic is the maximum insertion force for self-drilling/self-tapping and the results are summarised in Fig. 9a. It can be seen that the maximum insertion force for self-drilling/self-tapping can be reduced from 192 N for untreated pins to 182 N for the CCT treated pins. The maximum temperature recorded during the insertion period reduced from 31.2 ± 1.0 °C when using the untreated pins to 26.1 ± 0.5 °C (Fig. 9b). These data are mean values from 3 sets of pins to ensure reliability.

**Fig. 10:**
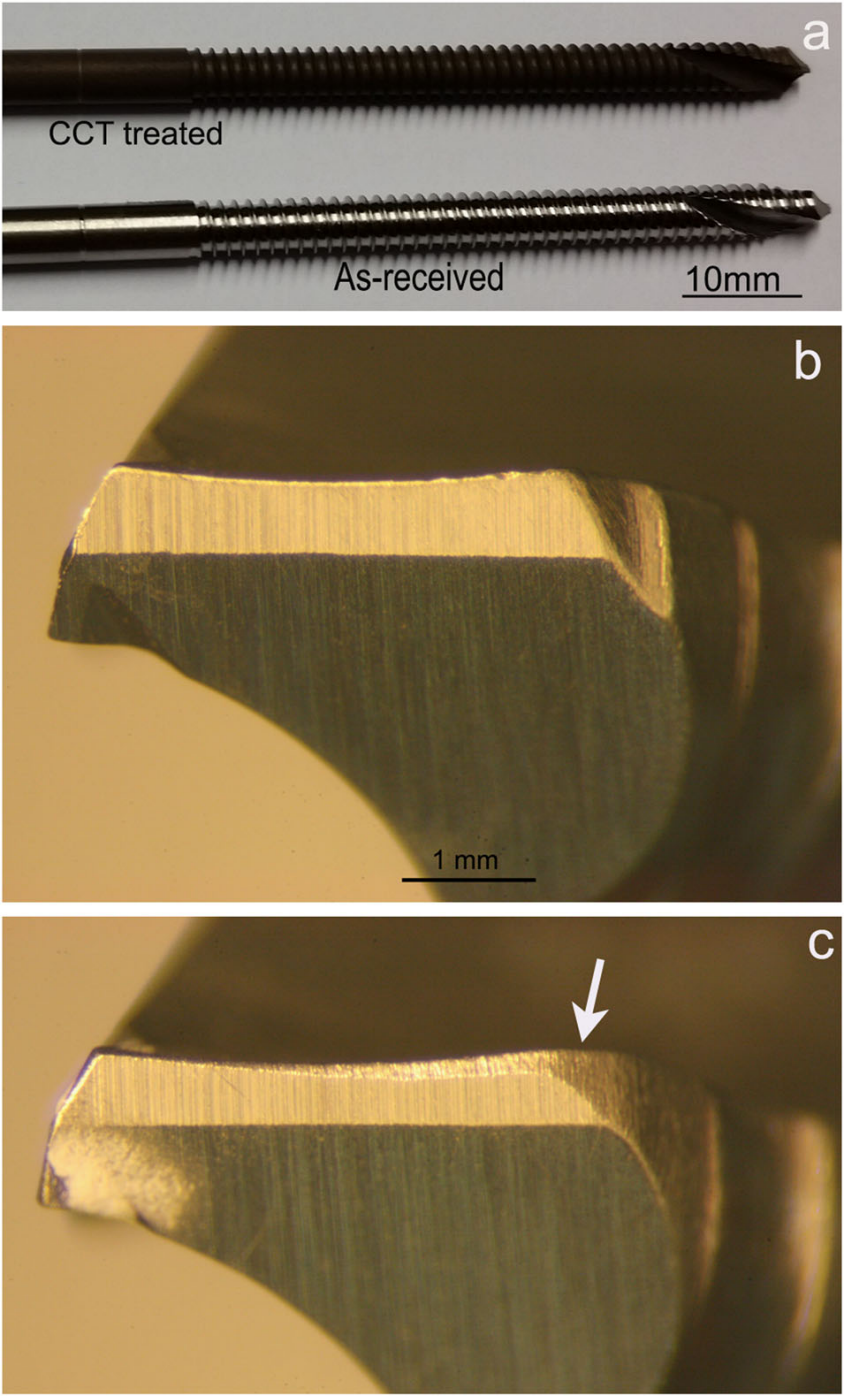
Optical images of **a** untreated and CCT treated self-drilling/self-tapping pin; untreated drilling blades **b** before and **c** after the insertion tests (The arrow denotes the wear of the cutting edge)

**Fig. 11:**
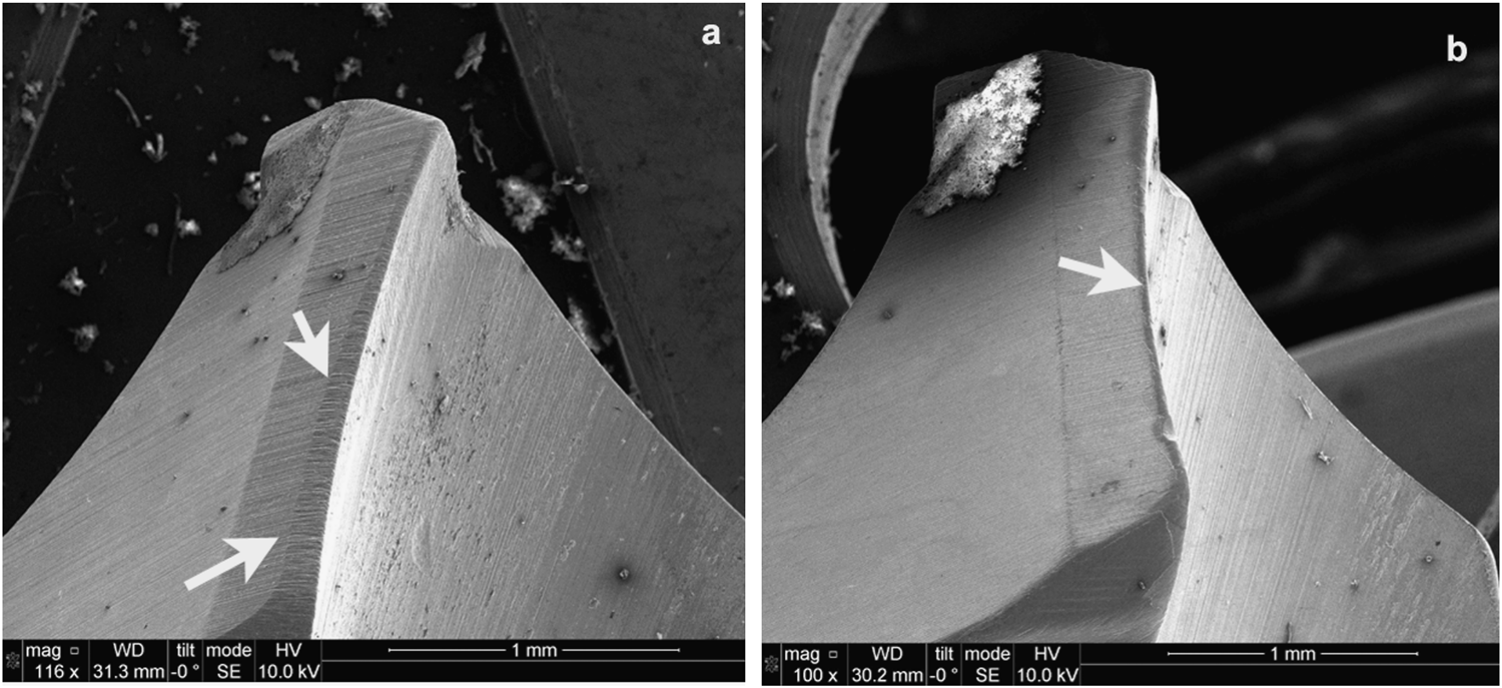
SEM images of the Ti pins after the insertion tests: **a** untreated and **b** CCT treated drilling blades (denoted by arrows)

**Fig. 12:**
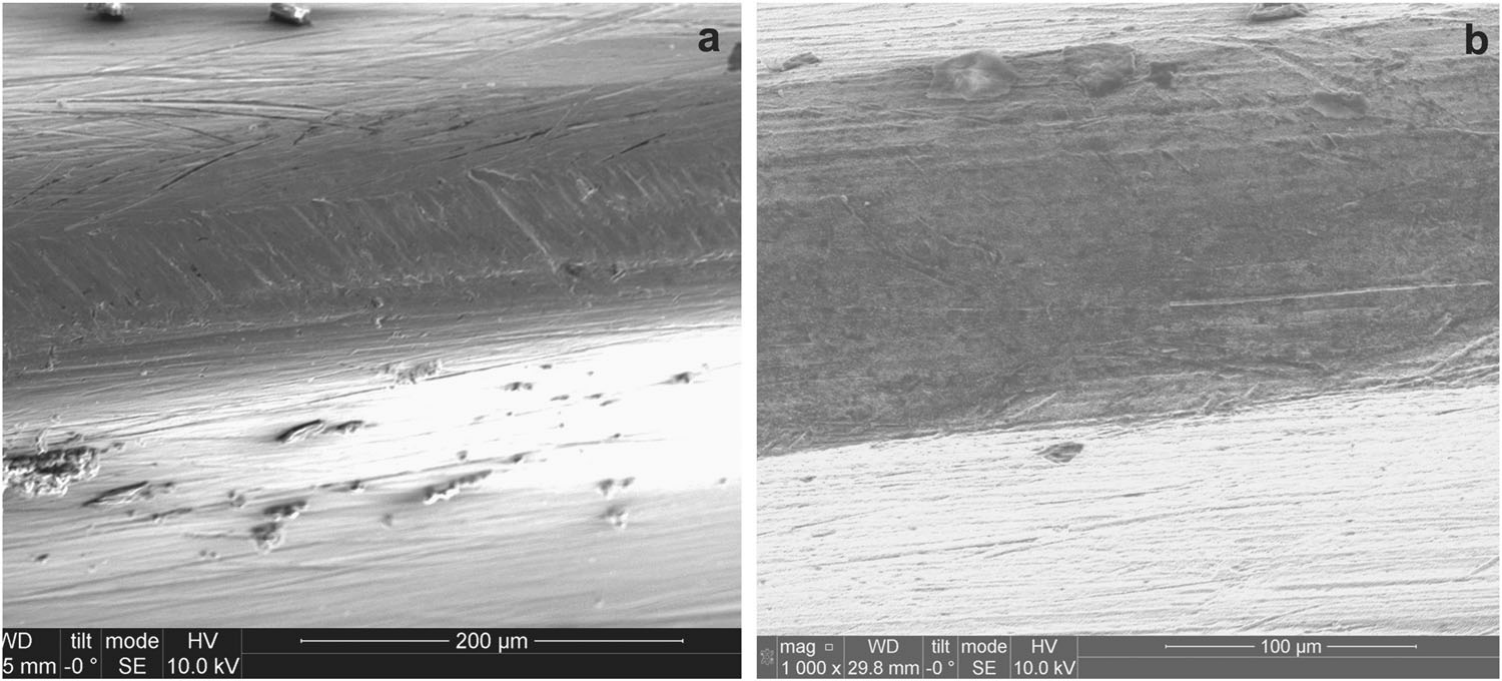
SEM images taken from the top of the Ti pin threads, **a** untreated and **b** CCT treated

Figure 10a shows the self-drilling/self-tapping pins before and after the CCT treatment. After the insertion tests, the drilling blades and the threads of the pin were observed using a Toolmakers microscope. It is clear that wear occurred to the drilling blades of the untreated pins after insertion test as evidenced by comparing the width of the cutting edge before (Fig. 10b) and after (Fig. 10c) the test; in contrast, no appreciable wear was observed from the cutting edge for the CCT treated pins after the insertion test under the Toolmakers microscope. This is further proved by SEM images in Fig. 11 where a flat strip at the edge of the drilling blade with parallel wear grooves can be clearly seen for the untreated pin (denoted by arrows in Fig. 11a) and severe abrasive wear occurred to the cutting edge of the blade during the insertion test. On the other hand, the cutting edge of the CCT treated pin is still sharp after testing (denoted by an arrow in Fig. 11b) without clear wear grooves under the given magnification. Similar results have also been obtained when examining other parts of the pins after the insertion test. For example, the top of all the threads on the untreated pins were worn as evidenced by the thick wear grooves (Fig. 12a) but only a few shallow wear grooves or scratches were observed on the top of the threads for the CCT treated pins after testing (Fig. 12b). Clearly, severe abrasive wear was observed on the tested untreated surface as evidenced by the dense and deep abrasive wear grooves. This is caused by the relatively short fibres within the bone substitute blocks although the overall hardness of the composite block (estimated to be 1.1 GPa based on the compressive strength) is lower than the hardness of untreated material (3.4 GPa). The different wear behaviour between the CCT treated and untreated pin surfaces could be attributed to the larger difference in hardness between the CCT treated (10.5 GPa) and the untreated (3.4 GP) surfaces. This is because the abrasive wear resistance of a material is inversely proportion to its surface hardness.

### Anti-bacterial efficacy

**Fig. 13:**
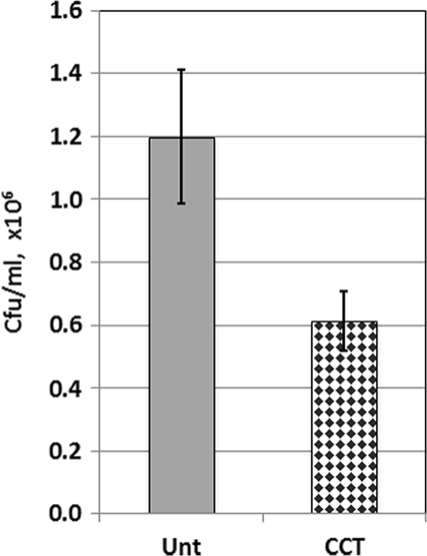
*S. aureus* recovered from fixation pins after 20 h incubation, following rinsing and sonication in 1.5 ml PBS. Results are expressed as cfu/ml × 10^6^ ± SD, (*n* = 4)

Sections cut from the untreated (Un Ti) and the ceramic conversion treated (CCT Ti) fixation pins were subjected to the antibacterial test procedure as described in Section 2.5, Fig. 1b. The control bacterial suspension contained about 7.6 × 10^8^ cfu/ml of *S. aureus* post-incubation. The experimental results revealed that viable counts of the bacteria recovered from the pins indicated that there was a significant reduction, *P* < 0.01 (0.0024), in the number of cfu recovered from the ceramic conversion treated pins (CCT Ti) compared to the untreated pins (Un Ti) of approximately 50 % (Fig. 13). This clearly indicates that the ceramic conversion can effectively improve the antibacterial efficacy of Ti6Al4V fixation pins.

## Discussion

### Improved biomechanics

As has been reported above, the ceramic conversion treatment can effectively reduce the insertion force (Fig. 9a) of Ti6Al4V fixation pins. This significantly improved bio-mechanical behaviour of the Ti6Al4V fixation pins by the ceramic conversion treatment should be attributed to the significantly improved bio-tribological properties of the titanium pins in terms of reduced friction and increased hardness. As has been clearly shown in Fig. 11, after inserting into the simulated cortical bone, severe wear has occurred to the cutting edge (as arrowed in Fig. 11a) as evidenced by the fact that the cutting edge become blunt with a platoon about 100 µm wide on the top; on other hand, the cutting edge of the CCT treated pins retains its original sharp edge with no appreciable wear (Fig. 11b).

The poor biomechanical and bio-tribological properties of Ti6Al4V pins are closely related to the low hardness, the electronic structure and crystal structure of titanium. This is because the hardness of the untreated Ti6Al4V is low (about 350HV) compared with hardened steel (about 700–800HV). Hence abrasive wear will occur when drilling relatively hard simulated cortical bone (Figs. 11a and 12a) since the abrasive wear resistance of a surface is proportional to its hardness [19]. In addition, it is also known that Ti has strong adhesive wear and galling tendency mainly due to its special electronic and crystal structure [20]. The experimental results reported in Section 3.2 have shown that the H/E ratio of the Ti6Al4V pins, which is a measure of the resistance to adhesive wear and galling, increased about 1.5 times after the CCT treatment.

As shown in Figs. 2–6, the ceramic conversion treatment can convert in-situ, the Ti surface into TiO_2_ rutile ceramic layer with a hardness of around 1050HV, which is about three times the hardness of the untreated material. Hence, it is expected both abrasive wear and adhesive wear of Ti could be reduced by the formation of the hard ceramic layer due to significantly increased hardness and the change of the nature of the surface from Ti to ceramic, respectively. In addition, this hard ceramic layer is in-situ converted and hence the concerns over the de-bonding or delamination of deposited ceramic coatings from the substrate can be addressed by the in-situ conversion approach. This has been proved by the scratch results shown in Fig. 7.

Most importantly, the untreated TiAl6Al4V is relatively soft and it has been widely reported that thin ceramic coating on a weak substrate can be easily damaged due to the well-known “thin egg-shell effect”—without sufficient mechanical support from the substrate, this hard coating will collapse [17]. In this research, TiAl6Al4V pins were ceramic conversion treated and in addition to the formation of a thin rutile layer on the top, oxygen also diffused into the subsurface (Figs. 3, 5), thus increasing the hardness (Fig. 6a) via solid solution hardening.

In short, it is the ceramic conversion induced surface hard ceramic layer supported by an oxygen hardened case beneath and the resulting significantly increased hardness, bonding strength and load supporting capacity of TiAl6Al4V pins that effectively improved the biomechanical and bio-tribological properties of TiAl6Al4V pins.

### Clinical indication and future work

The antibacterial properties demonstrated by the treated titanium pins could be very useful for reducing the high levels of pin site infections (as high as 30 %) seen following external fixation. This ranges from frank osteomyelitis to low grade infections which, nevertheless still cause loosening of pins at the bone-pin interface thereby compromising the structural rigidity of the fixation.

The antibacterial properties of TiO2, especially in the form of anatase following photoactivation with UV light, are well documented. Although most literature concerns the effects of anatase, rutile has a higher intrinsic photoactivation capacity and rutile nanoparticles showed a higher antimicrobial activity against E.coli [27]. The killing mechanism is thought to involve the production of destructive hydroxyl radicals, reactive oxygen intermediates and hydrogen peroxide. The reason for the antibacterial effect of our treated material in the absence of UV radiation is not yet entirely clear but studies have shown that photoactivation of both anatase and rutile can be achieved by short exposure to fluorescent light of wavelengths found in room lights [28]. Our results are consistent with those of a previous study (without UV irradiation) which showed that adhesion of the Gram positive bacterium *Streptococcus gordonii* was reduced by approximately 50 % on a rutile surface compared with anatase [29]. However the photoactivation effect depends on many factors including surface area, grain size and crystal dimensions [27, 28]. Independent of any possible photoactivation effect, bacterial adherence is dependent on several factors including surface area, surface energy and the coefficient of friction, which is reduced on the modified pin surface. The reason for reduced numbers of bacteria on our modified surface clearly merits further study with more bacterial strains, given the potential clinical applications.

By reducing the number of pin site infections, this surface treatment may reduce the need for antibiotics. This can be in the form of systemic antibiotics used to treat pin site infections or antibiotic impregnated coatings that have been developed for ex-fix pins. This would in turn reduce the problem of antibiotic resistance, which is currently a major issue facing the medical community. Moreover, as ceramic conversion essentially involves the oxidation of titanium it does not have the potentially toxic effects caused by the leaching of heavy metal ions from other coatings such as silver [8].

The reduced force needed to insert the ceramic conversion treated pins, caused by an increase in surface hardness, leads to a smaller rise in temperature in the adjacent soft tissues and thereby potentially causing less bone and soft tissue necrosis. This in turn provides less dead tissue around the pin sites which would otherwise be an excellent medium for bacterial colonisation. Furthermore, less necrotic bone surrounding the pin would help bio-integration and potentially lead to a lower incidence of pin loosening.

Future work could look at exploiting the inherent photocatalytic activity of titanium oxide. Titanium oxide surfaces are known to have increased antibacterial properties following exposure to UV radiation [10]. Titanium oxide surfaces are thought to kill bacteria by the breakdown of bacterial cell wall and membrane by reactive oxygen surfaces. UV irradiation of the surfaces causes an increase in free radicals thereby increasing the antibacterial effect. In addition, future work could look at the antibacterial effect for other Gram positive and Gram negative bacteria that are known to cause pin site infections such as *Staphylococcus epidermidis*, *Escherichia coli and Pseudomonas aeruginosa* [30].

Ultimately, the aim will be to see if there is a clinically demonstrable and significant benefit. To this end, we will first need to perform animal trials and if results are favourable, then to proceed to a randomised controlled trial. Finally, the ceramic conversion technique described is a very inexpensive treatment, making it a very commercially viable process compared to other surface treatments/coatings such as hydroxyapatite coatings which are prohibitively expensive.

## Summary and conclusions

In this study, an advanced surface engineering technology based on cost-effective and fully environmentally friendly CCT has, for the first time, been successfully applied to TiAl4V external fixation pins. This study demonstrates that the surface of TiAl4V external fixation pins can be converted into a TiO_2_ rutile layer (~2 μm in thickness), which is supported by an oxygen hardened case (~15 μm in thickness). Scratch testing revealed strong bonding between the surface rutile layer and the hardened case beneath due to the in-situ conversion nature. The surface hardness of Ti pins increased from 320 to 1050 HV following the CCT treatment.

When inserting into high density bone simulation material, the maximum insertion forces were reduced from 192 N when using the untreated pins to 182 N when the CCT treated pins were tested; the maximum temperature recorded during the whole insertion test was reduced from 31.2 °C for the untreated pins to 26.1 °C for the CCT treated pins. Post-testing SEM examination of the tested pins revealed that severe wear occurred to the cutting edge of the untreated Ti pins whilst no appreciable wear was observed for the CCT treated pins mainly due to the improved surface hardness by rutile layer, good bonding strength by in-situ conversion and enhanced load bearing capacity by the oxygen diffusion hardened case. The antibacterial test also revealed that the bacterial viable counts of the bacteria recovered from the pins indicated that there was a significant reduction, *P* < 0.01 (0.0024), in the number of cfu recovered from the ceramic conversion treated pins compared to the untreated pins of approximately 50 %.

The promising experimental results encourage the exploitation of the inherent photocatalytic activity of titanium oxide to further enhance the anti-bacterial efficacy of the CCT treated Ti pins. In addition, animal trials could be performed before proceeding to a randomised controlled trial.
